# Genetic Analysis of Vertebral Regionalization and Number in Medaka (*Oryzias latipes*) Inbred Lines

**DOI:** 10.1534/g3.112.003236

**Published:** 2012-11-01

**Authors:** Tetsuaki Kimura, Minori Shinya, Kiyosi Naruse

**Affiliations:** * Laboratory of Bioresources, National Institute for Basic Biology, Okazaki 444-8585, Aichi, Japan; † Genetic Strains Research Center, National Institute of Genetics, Mishima 411-8540, Japan; ‡ Department of Basic Biology, School of Life Science, Graduate University for Advanced Studies (SOKENDAI), Kanagawa 240-0193, Japan

**Keywords:** vertebral number, abdominal vertebrae, caudal vertebrae, genome-wide marker set

## Abstract

Vertebral number is the most variable trait among vertebrates. In addition to the vertebral number, the ratio of abdominal to caudal vertebrae is a variable trait. The vertebral number and the ratio of abdominal to caudal vertebrae contribute to vertebrate diversity. It is very interesting to know how to determine the vertebral number and the ratio of abdominal to caudal vertebrae. In this study, we identify differences in the vertebral number and the ratio of abdominal vertebrae to vertebral number between two inbred lines of medaka, namely, Hd-rRII1 and Kaga. To identify the genetic factor of those differences, we performed quantitative trait locus (QTL) analysis for vertebral number and the ratio of abdominal vertebrae to vertebral number using 200 F_2_ fish. Our results show a suggestive QTL of the ratio of abdominal vertebrae to vertebral number on chromosome 15, and five QTL of vertebral number on chromosomes 1, 10, 11, 17, and 23. The QTL on chromosome 15 contains *hoxDb* cluster genes. The QTL of vertebral number include some genes related to the segmentation clock and axial elongation. In addition, we show that the difference in vertebral number between two inbred lines is derived from differences in the anteroposterior length of somites. Our results emphasize that the developmental process should be considered in genetic analyses for vertebral number.

Vertebral number is the most variable trait among vertebrates. The adult frog has 6–9 vertebrae, whereas caecilian amphibians have 95–285 vertebrae (Richardson *et al.* 1998). These variations confer morphological diversity in vertebrates. Therefore, it is developmentally and evolutionarily interesting to elucidate the genetic factors underlying vertebral number. To date, developmental analysis has shown that vertebrae are derived from somites. The resegmentation model describes the vertebrae as structures that are developmentally derived from somites. According to the resegmentation model, each vertebra is formed through recombination of the anterior and posterior halves of two adjacent sclerotomes (Remak 1855; Bagnall *et al.* 1988; Saga and Takeda 2001). Somites are rhythmically formed from presomitic mesoderm as a periodic pattern along the anteroposterior axis of embryos. This process of somitogenesis is well explained by the clock and wavefront model (Cooke and Zeeman 1976; Dubrulle *et al.* 2001; Murray *et al.* 2011). Moreover, developmental analysis has shown that vertebral number is decided by a balance between the pace of the segmental clock and axial elongation (Gomez *et al.* 2008; Gomez and Pourquié 2009). The zebrafish mutant hes6, which carries one of the segmental clock genes, shows decreased vertebral number. Previous pig QTL analysis revealed that *NR6A1* and *vertnin* (*VRTN*) were underlying vertebral number (Mikawa *et al.* 2007, 2011), but the role of the genes in the segmental clock and axial elongation was unclear.

In addition to vertebral number, the ratio of abdominal to caudal vertebrae is a variable trait. The vertebral column at least is divided into two parts, namely, abdominal and caudal. Abdominal vertebrae are roughly defined as anterior rib-bearing structures, whereas caudal vertebrae are posterior structures that possess a hemal arch. The ratio of abdominal to caudal vertebrae has also been determined to be species-specific (Richardson *et al.* 1998; Ward and Brainerd 2007; Gomez and Pourquié 2009). Both the bronze featherback (*Notopterus notopterus*) and bichir (*Polypterus bichir*) have a long body, but whereas the bronze featherback has a long tail, the bichir has a long trunk (Ward and Brainerd 2007). In most species, the increase in the total number of vertebrae involves a parallel increase in the abdominal and caudal regions (Polly *et al.* 2001; Ward and Brainerd 2007). In fish, some species show an increase in the number of abdominal vertebrae, whereas others display an increase in the number of caudal vertebrae (Ward and Brainerd 2007). It is now generally accepted that vertebrate anteroposterior axial regionality is specified by the combined expression of the Hox genes known as the Hox cord (Kessel and Gruss 1990; Burke *et al.* 1995; Carapuço *et al.* 2005). Moreover, the anterior boundary of *hoxd12a* can be correlated with the boundary between abdominal and caudal vertebrae in zebrafish (van der Hoeven *et al.* 1996; Morin-Kensicki *et al.* 2002). Although *hoxd12a* is expressed in the boundary, the mechanism involved in defining this boundary is still unclear.

To elucidate genetic factors underlying vertebral number and the ratio of abdominal vertebrae to caudal vertebrae, we performed QTL analysis using medaka, a small, egg-laying freshwater fish from eastern Asia. More than 10 inbred strains of medaka are currently available (Hyodo-Taguchi 1996; Kinoshita *et al.* 2009). Because an inbred strain is genetically uniform, genetic factors are easily distinguishable from environmental factors. In addition, numerous polymorphisms in the genotype and phenotype of the medaka have been observed among inbred lines. As another advantage, the medaka lays around 20 eggs every morning, and therefore, several hundred progeny from the same parents can be easily obtained for research investigations. The medaka is currently emerging as an important vertebrate model (Wittbrodt *et al.* 2002; Takeda and Shimada 2010). A high-quality draft of the medaka genomic sequence is available (Kasahara *et al.* 2007), making it possible to conduct comparative analyses of specific candidate regions with other species and to subsequently narrow down gene regions of interest. Thus, it is ideal to use the medaka as an animal model for the elucidation of genetic factors underlying complex traits (Kimura *et al.* 2007).

In this study, we have shown differences between the vertebral number and the ratio of abdominal vertebrae to vertebral number between the Hd-rR-II1 strain and the Kaga strain. We have also performed QTL analyses for those traits by using 200 F_2_ progenies.

## Materials and Methods

### Fish strains

The vertebral number in fish is influenced by temperature during its ontogeny (Hubbs 1940; Gabriel 1944). We incubated all eggs at 28° until hatching to minimize the relative effects of temperature. The medaka adults were maintained at 26° on a 14-hr light/10-hr dark cycle. Hd-rR-II1 and Kaga strains (Hyodo-Taguchi 1996; Naruse *et al.* 2004a) are inbred medaka strains established from a southern and a northern Japanese population, respectively (Takehana *et al.* 2003). Two pairs of Hd-rR-II1 and Kaga strains were crossed to generate the F_1_ progeny. Sex combination was reciprocal. A total of 200 F_2_ progeny were obtained from intercrossing 11 pairs of the F_1_ progeny.

### Skeletal preparation and vertebral count

After four months, fish were killed. We removed the eyeballs and fixed them in 100% ethanol for DNA extraction. Fish were fixed in 3.7% formaldehyde/PBS overnight and washed with water. After the scales were removed, the samples were rinsed with 0.5% potassium hydroxide (KOH) solution and stained with ethanol-saturated 20% alizarin red S/0.5% KOH for 1–2 days. After staining, the samples were washed in water and then transferred to 20% glycerol containing 0.1% hydrogen peroxide to bleach any pigments. The samples were then dehydrated using an ascending glycerol series.

**Figure 1:**
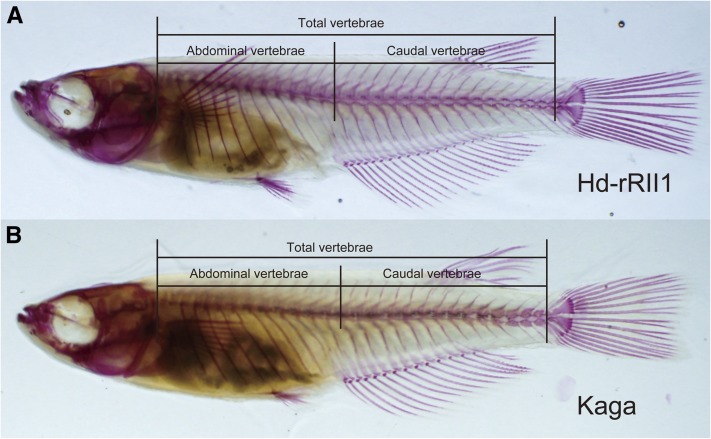
Vertebral number differs between Hd-rdII1 and Kaga strains. Photographs are adult medaka skeletal preparations of Hd-rRII1 (A) and Kaga (B). The lines show the ranges of total vertebral number, abdominal vertebrae, and caudal vertebrae. We divided vertebrae into the abdominal and the caudal according to Iwamatsu (1997). (A) The fish has 13 abdominal vertebrae and 16 caudal vertebrae, for a total of 29 vertebrae. (B) The fish has 13 abdominal vertebrae and 14 caudal vertebrae, for a total of 27 vertebrae. When we counted the vertebrae number, urostyle was excluded from the vertebrae.

We counted the number of vertebrae in the images of prepared skeletal samples according to a study by Iwamatsu (1997). Abdominal vertebrae were defined as the vertebrae running from the atlas to edge of the rib cage (Figure 1). Caudal vertebrae were defined as structures running from the first hemal spine to the first preural centrum. The urostyle was excluded in the analysis. The differences between Hd-rR-II1 strain and Kaga strain were examined using Welch’s *t*-test. Correlations among the vertebral number, abdominal vertebrae, and caudal vertebrae were calculated using Spearman’s rank correlation coefficients in the R software.

### Somite length and trunk length measurement

To measure the anteroposterior length of two to four somites, four to six somite embryos were photographed in 2% methylcellulose using a Leica M125 stereomicroscope equipped with an Olympus DP71 universal camera. To measure the trunk length, an egg envelope from a 7-day-old embryo was removed using a hatching enzyme and then photographed. Measurement was performed using the ImageJ software.

### Genotyping

We designed 147 PCR product length polymorphism (PLP) primers, as described by Kimura and Naruse (2010), for QTL analysis (supporting information, Table S1).

Genomic DNA was extracted from fish eyeballs fixed in 100% ethanol. The samples were suspended in 500 µl of lysis buffer (10 mM Tris-HCl, pH 8.0; 1 mM EDTA; 200 μg/ml proteinase K) and incubated at 55° for 3 hr. Thereafter, the samples were incubated at 95° for 10 min, after which 500 µl of DNase-free water was added. All genomic DNA samples were stored at −20° until use.

PCR reactions were performed as described previously (Kimura and Naruse 2010). The protocol was modified to use KAPATaq Extra DNA polymerase (Kapa Biosystems, Woburn, MA). PCR products were analyzed by using a DNA-500 kit on MCE-202 MultiNA (SHMADZU, Kyoto, Japan). In total, 200 F_2_ fish were genotyped and used in QTL analysis (File S1).

### QTL analysis

A linkage map was constructed by using Mapmaker/EXP 3.0b (Lander *et al.* 1987). Recombination frequencies were converted to map distance (cM) by using Haldane’s map function. A logarithm of the odds score of 3.0 was used to determine the order of the loci. After the ordering of markers within each linkage group, the RIPPLE command allowed the testing of robustness of the map obtained.

QTL analysis was performed using R/qtl (Broman *et al.* 2003; Broman and Sen 2009). Nonparametric interval mapping is performed with the scanone function, using the argument “model = np.” The logarithm of odds (LOD) significance threshold for interval mapping was determined by 10,000 iterated permutations. Two-QTL scans were performed using Haley-Knott regression (Haley and Knott 1992). The LOD score significance thresholds for two-QTL scans were determined by using 1000 iterated permutations. The genome-wide significance thresholds were set at α = 0.05. The bayesint function was used to calculate the Bayes credible interval (95%) for interval estimation of QTL location. The fitqtl function was used to calculate the percentage variance explained by the peak marker for each QTL.

## RESULTS AND DISCUSSION

### Genetic linkage map

A total of 147 PLP markers based on the medaka genome sequence were developed, from which a linkage map was constructed on the basis of the data generated from 200 F_2_ progeny. The map had 24 linkage groups (same number as the number of chromosomes), and the total length of the map was estimated at 1493.7 cM, with the average distance between markers calculated as 12.1 cM (Table S1). Our map shows almost the same length as previous maps (Naruse *et al.* 2004b; Kimura *et al.* 2005), and thus, we concluded that this map is sufficient for QTL analysis. Because our marker set covers 700 M bases, 1 cM corresponds to 469 K base pairs.

### Vertebral counts

To identify the genetic factors controlling vertebral number in the medaka, we compared the vertebral number of Hd-rR-II1 with Kaga (Table 1). The significant difference was observed in average of vertebral number using Welch’s *t*-test (*P* = 7.5 × 10^−30^). Hd-rR-II1 strain has a larger vertebral number than does the Kaga strain. Because previous publications reported that, in medaka, an average of the vertebral number of laboratory strains and wild populations were 30 vertebrae (Ogawa 1971; Ali and Lindsey 1974; Yamahira and Nishida 2009), the vertebral number of Kaga strain was the lowest number of medaka. Because all loci were shown to be homozygous, it was relatively easy to show that the recessive traits observed in the inbred lines were hidden in the wild population and that extreme phenotype could actually appear in the inbred lines. To generate an F_1_ progeny, we crossed Hd-rR-II1 and Kaga strains reciprocally. The F_1_ progeny showed almost the same vertebral number as the Hd-rR-II1 strain, although the mean value shifted downward (Table 1). The vertebral numbers show a greater degree of variation in the F_2_ progeny than in the three isogenic populations (Hd-rR-II1, Kaga, and F_1_). The range of the F_2_ fish traits ranged from those observed in the Kaga strain to those observed in the Hd-rR-II1 strain (Table 1).

**Table 1:** Vertebral number and the ratio of abdominal vertebrae

	Vertebral Number						Mean of Ratio of Abdominal Vertebrae	Total Number of Individuals
Strain	26	27	28	29	30	Mean		
Hd-rRII1	−	−	−	24	10	29.32 ± 0.08	0.443 ± 0.002	34
Kaga	1	33	1	−	−	27.00 ± 0.04	0.453 ± 0.003	35
F_1_	−	−	1	57	−	28.98 ± 0.02	0.421 ± 0.002	58
F_2_	−	13	77	91	19	28.60 ± 0.05	0.442 ± 0.001	200

Vertebral number was counted in four-month-old adult fish stained with alizarin red S. The Vertebral Number column reports distribution and mean ± SEM of the vertebral number. For example, in the Hd-rRII1 strain, 24 fish had 29 vertebrae, and 10 fish had 30 vertebrae. The mean ± SEM of the ratio of abdominal vertebrae to total vertebrae is also reported. All differences between Hd-rRII1 and Kaga strains showed significance based on Welch’s *t* test.

Previous studies using the medaka suggested that the number of abdominal and caudal vertebrae were influenced by different genetic factors (Ali and Lindsey 1974; Yamahira and Nishida 2009; Yamahira *et al.* 2009; Kiso *et al.* 2011). And the vertebral number can increase independently in caudal and abdominal regions (Ward and Brainerd 2007). To estimate a relation among the vertebral number, abdominal vertebrae, and caudal vertebrae, we calculated the correlations. The correlation between the number of abdominal and caudal vertebrae was negative in all generations; the correlation coefficients were −0.40, −0.83, −0.96, and −0.30 for Hd-rR-II1, Kaga, F_1_, and F_2_, respectively. The correlation between the number of abdominal vertebrae and total vertebral number was not consistent among all the generations; the correlation coefficients were 0.00, 0.28, −0.06, and 0.42 for Hd-rR-II1, Kaga, F_1_, and F_2_, respectively. The correlation between the number of caudal vertebrae and total vertebrae was positive in all the generations; the correlation coefficients were 0.91, 0.28, 0.33, and 0.71 for Hd-rR-II1, Kaga, F_1_, and F_2_, respectively. In the F_2_ progeny, the negative correlation (between the number of the abdominal and caudal vertebrae) and the positive correlations (between the total vertebral number and the number of both the abdominal and caudal vertebrae) suggested that although both abdominal and caudal vertebrae were able to contribute to the increase in the number of vertebrae, the caudal vertebrae had contributed more. No traits were correlated with sex in the parental strains.

### QTL analysis of the ratio of abdominal vertebrae to vertebral number

Because F_2_ fish have varying numbers of vertebrae, we considered the ratio of abdominal vertebrae to total vertebral number as a trait. We found that the ratio between Hd-rR-II1 and Kaga strains was significantly different (judged by Welch’s *t*-test; *P* = 0.004) (Table 1). We then performed QTL analysis to elucidate the genetic factors underlying this trait.

**Figure 2:**
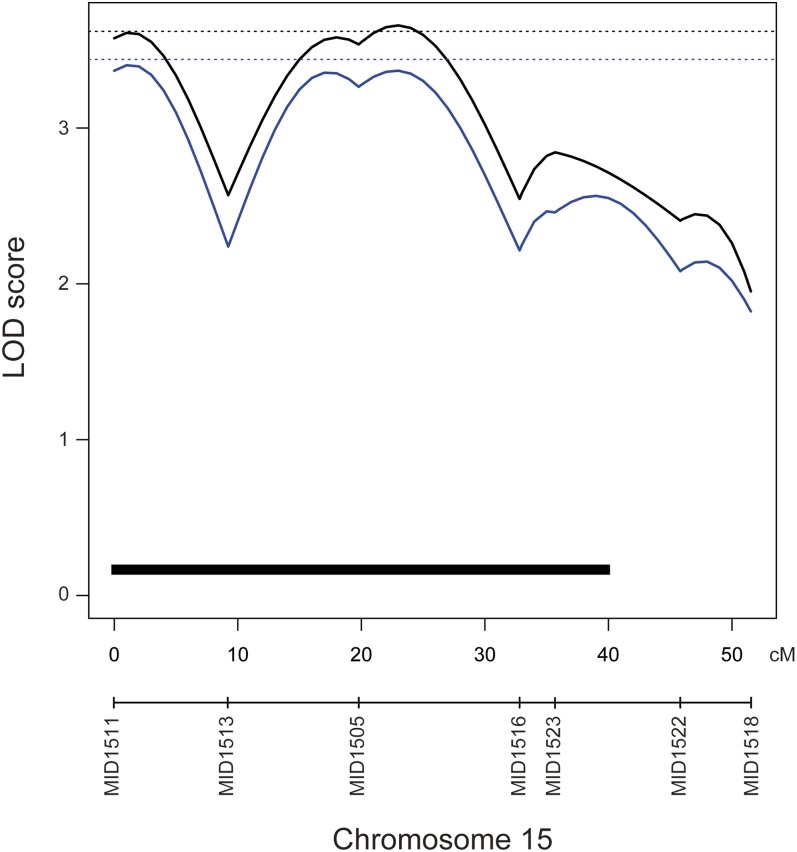
QTL associated with the ratio of abdominal vertebrae to vertebral number. Black and blue lines indicate the logarithm of odds (LOD) curves of interval mapping for the parametric and nonparametric analyses, respectively. The significance LOD threshold was determined by 10,000 iterated permutations. The black dashed line indicates genome-wide significance for the vertebral number at threshold α = 0.05 (LOD = 3.62). The blue dashed line indicates genome-wide significance for the ratio of the number of abdominal vertebrae at threshold α = 0.05 (LOD = 3.44). The black bar indicates Bayes credible interval (95%).

Because the distribution of the phenotype was not normal, we performed both parametric and nonparametric QTL analysis (Kruglyak and Lander 1995) (Figure 2 and Table 2). In the parametric analysis, we identified one significant QTL on chromosome (Chr) 15. The LOD peak of the QTL was 3.65, and the genome-wide LOD score significance threshold was 3.62. In the nonparametric analysis, we were not able to detect significant QTL. The genome-wide LOD score significance threshold was 3.44 (α = 0.05) and 3.10 (α = 0.1), and the highest LOD score was 3.40 on Chr15. Because this method extends rank-based test statistics (extension of Kruskal-Wallis test), this analysis does not work well in the case of many ties in the phenotypes. Therefore, the power of the nonparametric analysis was decreased in our case. However, as QTL on Chr15 was not detected by nonparametric analysis, we thought it was suggestive QTL. Subsequent analyses were parametric analyses. The Bayes credible interval suggested that the QTL spanned 40 cM, which corresponds to 18.8 Mb, located between markers MID1511 and MID1522. Contrary to the expectations from the parental strain phenotype, the Kaga allele from the QTL was associated with a decrease in the ratio of abdominal to total vertebrae. The QTL reflected the 7.9% phenotypic variance observed. We did not detect any QTL that showed an association with epistatic interactions in this analysis, possibly because of the very small sample size employed for the detection.

**Table 2:** QTL position and effect

		Position		LOD Score		Threshold						
Trait	Chr	Parametric	Nonparametric	Parametric	Nonparametric	Parametric	Nonparametric	Closest Marker	PVE	Hd-rRII1	Heterozygous	Kaga
Vertebral number	1	38.0	39.0	4.44	4.26	3.59	3.49	MID0122	2.6	28.96 ± 0.10	28.43 ± 0.08	28.49 ± 0.10
Vertebral number	10	8.0	7.0	14.57	12.81	3.59	3.49	MID1025	22.3	28.84 ± 0.10	28.86 ± 0.06	27.98 ± 0.08
Vertebral number	11	51.0	48.0	5.89	5.07	3.59	3.49	MID1116	7.6	28.94 ± 0.10	28.60 ± 0.07	28.16 ± 0.11
Vertebral number	17	40.0	40.0	4.28	4.11	3.59	3.49	MID1718	5.4	28.51 ± 0.10	28.46 ± 0.07	29.02 ± 0.11
Vertebral number	23	34.2	34.1	4.39	4.46	3.59	3.49	MID2313	4.6	28.77 ± 0.10	28.71 ± 0.07	28.20 ± 0.10
Ratio of abdominal vertebrae to vertebral number	15	23.0	1.0	3.66	3.40*^a^*	3.62	3.44	MID1505	7.9	0.449 ± 0.002	0.442 ± 0.002	0.434 ± 0.003

Threshold indicates the LOD score for a genome-wide significance threshold of α = 0.05 determined by 10,000 iterated permutations. Position indicates QTL peak position on chromosome (cM). The mean ± SEM of the phenotype of F_2_ fish for the different three genotypic classes of closest marker, Hd-rR-II1, heterozygous, and Kaga, are shown. The PVEs and means are values of parametric analysis. Chr, chromosome; LOD, logarithm of odds; PVE, percentage variance explained.

a Suggestive QTL.

It is well established that the anterior boundaries of *Hox* gene expression are concordant with some morphological boundaries established in vertebrates (Burke *et al.* 1995). In zebra fish, *hoxd12a* is expressed around the boundary of abdominal and caudal vertebrae (van der Hoeven *et al.* 1996; Morin-Kensicki *et al.* 2002). In medaka, *hoxD12a* has been mapped to Chr21, and thus, we did not detect any QTL encompassing *hoxD12a*. However, *hoxDb* cluster genes, *hoxD4b* and *hoxD9b*, are located underneath the QTL on Chr15 (Kurosawa *et al.* 2006). Previous studies showed that heritability also varies between abdominal and caudal vertebrae in the medaka (Yamahira *et al.* 2009; Kiso *et al.* 2011). Additionally, the effect of temperature also varies between abdominal and caudal vertebrae (Ali and Lindsey 1974; Yamahira and Nishida 2009; Yamahira *et al.* 2009; Kiso *et al.* 2011). These observations suggest that the ratio can vary independently of the length of the abdominal region. Because the caudal vertebrae had stronger correlation to vertebral number than did the abdominal vertebrae, we considered that the QTL on Chr15 was responsible for the length of the caudal region. Because the medaka grows continuously throughout its life cycle, it would be difficult to establish a suitable standard of length for this type of analysis.

### QTL analysis for total vertebral number

**Figure 3:**
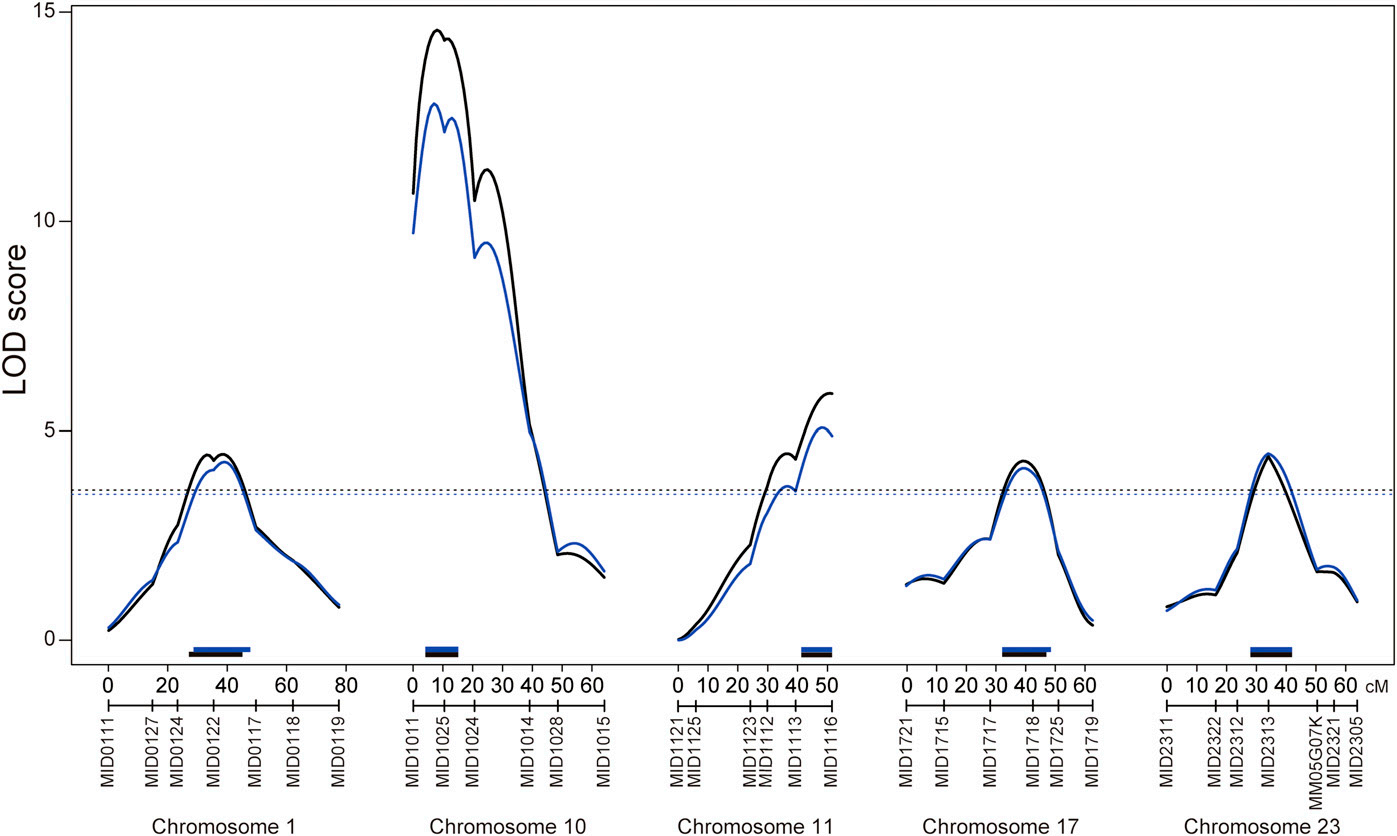
QTL associated with the vertebral number. Black and blue lines indicate the logarithm of odds (LOD) curves of interval mapping for the parametric and the nonparametric analyses, respectively. The significance LOD threshold was determined by 10,000 iterated permutations. The black dashed line indicates genome-wide significance for the parametric analysis at threshold α = 0.05 (LOD = 3.59). The blue dashed line indicates genome-wide significance for the nonparametric analysis at threshold α = 0.05 (LOD = 3.49). The black and blue bars indicate Bayes credible interval (95%) for the parametric and nonparametric analyses, respectively.

Because the distribution of the phenotype was not normal as the total vertebral number, we performed both parametric and nonparametric QTL analysis. In the parametric analysis, five chromosomes, namely, Chr1, Chr10, Chr11, Chr17, and Chr23, showed LOD scores higher than the genome-wide LOD score of 3.65, which was the significance threshold determined by permutation testing (Figure 3 and Table 2); the LOD peaks of these QTL were 4.44, 14.57, 5.89, 4.28, and 4.39, respectively. In the nonparametric analysis, we detected same five QTL (Figure 3 and Table 2). The LOD peaks of these QTL were 4.26, 12.81, 5.07, 4.11, and 4.46, and the genome-wide LOD score significance threshold was 3.49. In both analyses, the interval of QTL deduced by Bayes credible interval was same at three QTL on Chr10, Chr11, and Chr23, the QTL interval on Chr1 shifted but still overlapped, and the QTL interval on Chr17 was larger in nonparametric analysis. The Bayes credible interval suggested that the QTL on Chr1 spanned 18 cM (27–45 cM in parametric) and 19 cM (28–47 cM in nonparametric), which corresponds to 8.4 and 8.9 Mb of genomic region and that the QTL were located between markers MID0124 and MID0117. The QTL on Chr10 spanned 11 cM, which corresponds to 5.2 Mb of genomic region, and were positioned between markers MID1011 and MID1024. The QTL on Chr11 spanned 10.6 cM, which corresponds to 5.0 Mb of genomic region, and were located between markers MID1113 and MID1116. The QTL on Chr17 spanned 15 cM (32–47 cM in parametric) and 16 cM (32–48 cM in nonparametric), which corresponds to 7.0 and 7.5 Mb of genomic region, and were positioned between markers MID1717 and MID1725. The QTL on Chr23 spanned 14 cM, which corresponds to 6.6 Mb of genomic region, and were located between markers MID2312 and MM05G07K (Figure 3). The result of parametric analysis was concordant with the result of nonparametric analysis. Thus, we concluded that five QTL were actually significant.

To elucidate the effects of QTL on phenotype, we calculated the averages based on the genotype of the nearest markers of the LOD peaks (Table 2). Except for the QTL on Chr17, the Kaga alleles showed a decrease in the number of vertebrae, as would be expected from the parental strain phenotype. The two-QTL scan did not detect any statistically significant epistatic interactions.

Previous studies detected QTL for vertebral number using pig and rainbow trout (Nichols *et al.* 2004; Mikawa *et al.* 2007, 2011). Although one QTL was identified for vertebral number on OC6 in the rainbow trout, it is difficult to compare this data with our QTL because the genomic information of rainbow trout is not as complete as it is for other species. Another previous QTL study comparing wild boar with pig showed two genes, *NR6A1* and *VRTN*, that showed an association to an increase in vertebral number (Mikawa *et al.* 2007, 2011). However, this study did not detect any QTL on the chromosomes that contained these genes (Chr9 for *NR6A1* and Chr24 for *VRTN* in the medaka according to Ensembl Genome Browser). It is possible that any variations observed in our inbred lines were not derived from these genes.

Previous studies also showed that axial elongation is generally associated with an increase in vertebral number (Wake 1966; Lindsey 1975; Richardson *et al.* 1998). On the other hand, Schröter and Oates (2010) showed that in the zebrafish, the anteroposterior somite length was associated with anteroposterior vertebral length, suggesting that differences in vertebral number may be attributable to changes in axial elongation or in the segmentation clock. Therefore, genes related to the axial elongation process and the segmentation clock genes can possibly serve as causative genes influencing the total vertebral number in a species (Gomez and Pourquié 2009). It is well known that FGF, NOTCH, and WNT signaling pathways play a crucial role in the axial elongation and the segmentation clock (Saga and Takeda 2001; Dubrulle *et al.* 2001; Murray *et al.* 2011). We investigated whether those candidate genes would be contained to the QTL regions using BioMart Version 0.8. The QTL region on Chr1 included three candidate genes, *deltex homolog 4*, *secreted frizzled-related protein 2*, and *axin 1*. The *deltex homolog 4* is the NOTCH signaling gene. The *secreted frizzled-related protein 2* and *axin 1* are the WNT signaling genes. The QTL region on Chr10 included four candidate genes. The *fgf13* and *fibroblast growth factor receptor-like 1* are FGF signaling genes and *shisa homolog 3* is an antagonist of FGF and WNT signaling. The *transforming growth factor beta 2* expressed in zebrafish notochord is also a candidate gene in this QTL. The QTL region on Chr11 included two candidate genes, *hey1* and *Brachyury/notail*. The *hey1* is NOTCH signaling gene. The *Brachyury/notail* is a transcriptional factor having an important role in mesoderm differentiation. The QTL region on Chr17 included four candidate genes, *hes6*, *snail2*, *gbx1*, and *wnt3a*. The *hes6* is one of the segmental clock genes, and the zebrafish *hes6* mutant has an enlarged somite (Schröter and Oates 2010). The *snail2* and *gbx1* are WNT signaling genes. Additionally, wnt3a is the ligand of the signaling, and mouse mutants of *Wnt3a* lack caudal somites (Takada *et al.* 1994). The QTL on Chr23 included three candidate genes, *wnt5b*, *prickle homolog 1*, and *fgf6*. The *wnt5b* is ligand of WNT signaling. The *prickle homolog 1* is WNT signaling gene. The *fgf6* is a ligand of FGF signaling and expresses in zebrafish notochord.

**Figure 4:**
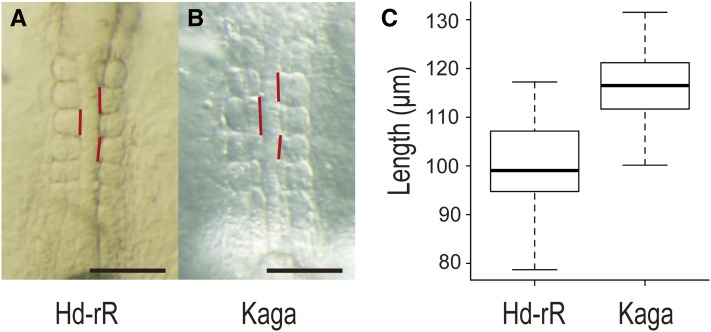
Anteroposterior length of somites two to four differs between Hd-rRII1 and Kaga strains. Anteroposterior length of somites two to four was measured in stage 20–21 embryos (four to seven somite stage). (A) Five somite stage Hd-rRII1 embryo. (B) Five somite stage Kaga embryo. Vertical red lines show anteroposterior length of each somite. The scale bars represent 100 µm. (C) Box plot of anteroposterior length of somites two to four. The mean ± SEM of the anteroposterior length of somites two to four are 99.6 ± 1.5 in Hd-rRII1 (n = 34) and 116.8 ± 1.4 in Kaga (n = 33). Kaga strain has approximately 17% longer somites than Hd-rRII1. The somite length showed significant difference based on Welch’s *t*-test (*P* = 2.64 × 10^−12^).

Because the zebra fish *hes6* mutants possess a slower segmentation clock and a lower number of vertebrae compared with the wild type, we measured the anteroposterior somite length of Hd-rR-II1 and Kaga embryos (Figure 4, A and B). As expected, Kaga showed a smaller number of vertebrae, yet with 1.18-fold longer somites than those in Hd-rR-II1 (Figure 4C). However, if both strains possessed the same body length, Kaga should have 25 vertebrae, as inferred from the ratio of somite length, but it actually consisted of 27 vertebrae. Additionally, Kaga is shorter than Hd-rR-II1, contrary to the anticipation at hatching stage (Figure S1). Thus, somite length may explain the difference in vertebral number among the inbred lines, but the results of this study suggest that it is not the only factor.

In this study, we detected one suggestive QTL associated with the ratio of abdominal vertebrae to total vertebral number and five QTL associated with vertebral number. Additionally, we found that the anteroposterior length of somites explains the difference in the total vertebral number among the inbred lines. Thus, the developmental process provides important clues for genetic analysis. The identification of genes within these QTL can help in defining mechanisms underlying the establishment of vertebrate body plans.

## Supplementary Material

Supporting Information
