# Supplementary material for: Genetic Analysis of Vertebral Regionalization and Number in Medaka (Oryzias latipes) Inbred Lines
Source: G3 (Bethesda). 2012 Nov 1;2(11):1317–23. doi: 10.1534/g3.112.003236 (PMC3484662; doi:10.1534/g3.112.003236)
Supplement: Supporting Information [file supp_2_11_1317__index.html]

Supporting Information 

# Genetic Analysis of Vertebral Regionalization and Number in Medaka (*Oryzias latipes*) Inbred Lines

## Supporting Information for Kimura, Shinya, and Naruse, 2012

**Files in this Data Supplement:**

- Supporting Information - Figure S1, File S1, and Table S1 (PDF, 1 MB)
- Figure S1 - Measure of fry of Hd-rRII1 and Kaga (PDF, 1 MB)
- Table S1 - Marker information (PDF, 104 KB)
- File S1 - Input data (.xls, 450 KB)
