# Supplementary material for: Genetic Analysis of Vertebral Regionalization and Number in Medaka (Oryzias latipes) Inbred Lines
Source: G3 (Bethesda). 2012 Nov 1;2(11):1317–23. doi: 10.1534/g3.112.003236 (PMC3484662; doi:10.1534/g3.112.003236)
Supplement: Supporting Information [file supp_2.11.1317_003236SI.pdf]

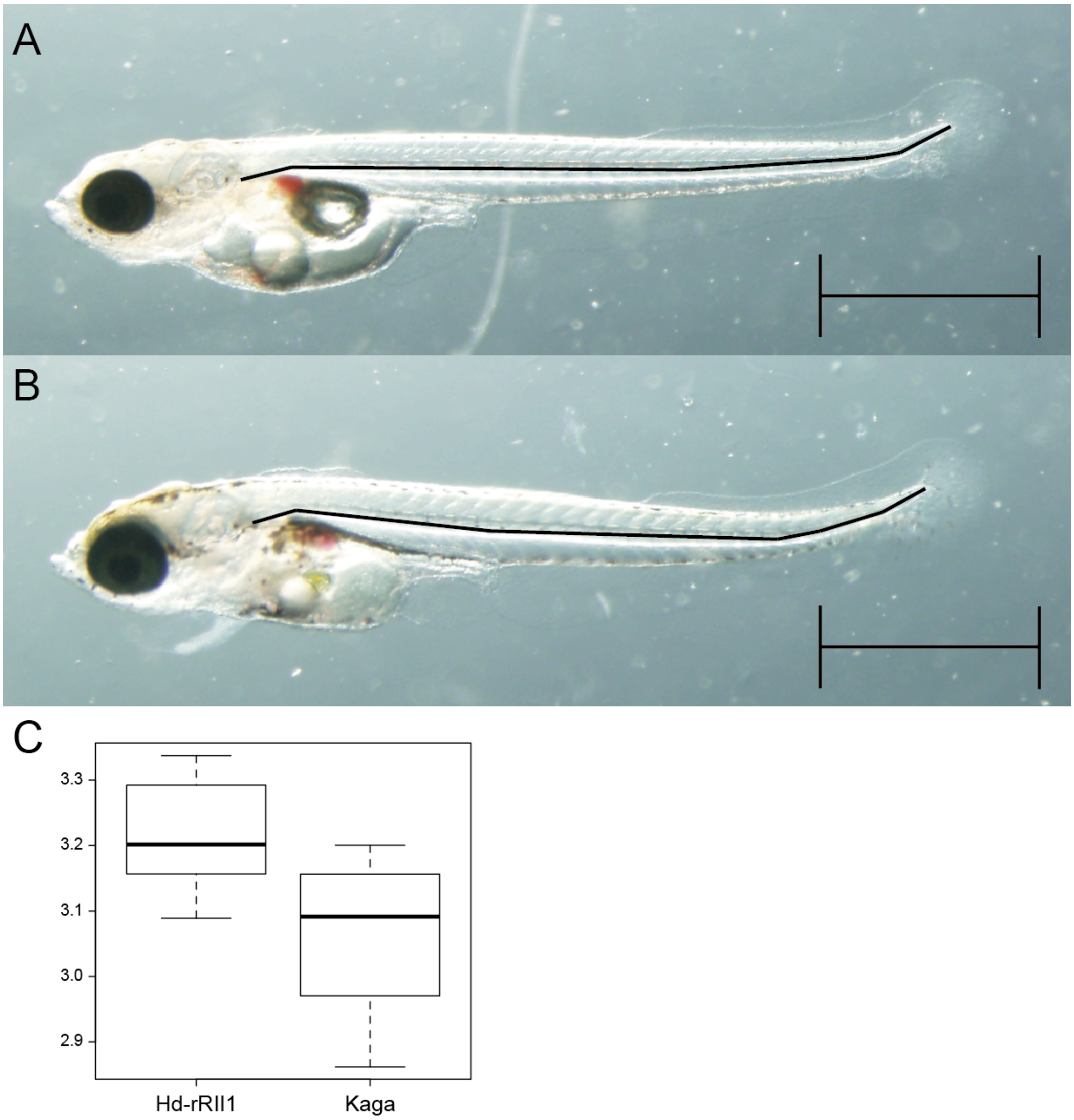

Figure S1 Measure of fry of Hd-rRII1 and Kaga. Photo is seven days fry of Hd-rRII1 (A) and Kaga (B). The scale bars represent 1 mm. Straight line was drawn along with notochord and it was considered as trunk length. The length was measured in imageJ software. (C) Boxplot of the trunk length. The mean ± S.E.M. of the trunk length of are 3.22 ± 0.02 mm in Hd-rRII1 (n = 16), and 3.06 ± 0.04 mm in Kaga (n = 11), respectively. Hd-rRII1 is longer than Kaga in this stage. The trunk length showed significant difference as judged by Welch's *t* test (p = 0.002).

File S1

Input Data

Avaialble for download at http://www.g3journal.org/lookup/suppl/doi:10.1534/g3.112.003236/-/DC1.

Table S1 Marker information

Marker	Chromosome	Position (cM)	Primer1	Primer2	Amplicon size (bp)	
					Hd-rRII1	Kaga
MID0111	1	0.0	TGCTAACATTTTCTTTGCATCTAAAG	GCACCACCATAGCCAACCT	150	135
MID0127	1	14.8	AATGAAGACCAGAACATGTTTCTAAAT	TGTAGTTATTGCATTGCCTAATTTAAT	147	130
MID0124	1	23.2	TCAGTCCCAAATGGCTTAAAAC	AATCTGCTTCTCATTTCTTCAGTG	148	134
MID0122	1	35.3	CATAAGTAACTTTCATACACTCATGATTAA	TAGCGAGACGATTTTGGTCC	103	91
MID0117	1	49.5	GGCCCTTTGAGGACAGCC	AAAACATAAAGTGTAATCTTTTAAAAAGTCC	157	131
MID0118	1	62.0	GAGTCTTGAGACTGATGGCAGC	CGCCCTGCTCCGGCCTCG	208	132
MID0119	1	77.5	GGTCTTTGTACACATTCCTGTGAG	CAGAGGTAGACATGCCTTGATATG	143	121
MID0211	2	0.0	GCTGCTGCGTTCTGAACAA	AACTTTGTTGGAATCACTGTTCTCC	125	104
MID0221	2	20.6	CTGTCATTCCTTCAAATGCTG	ACCGGAAAGGACAAAGGTC	100	93
MID0229	2	30.7	CCAACAAAACACCACCGTC	CGTAATGGACATTTTAGAACTTCC	164	142
MID0215	2	39.8	AGTAGGCACACAAAATGTTCGG	CGCCGGCGTTCAGGTGGG	120	111
MID0216	2	54.7	CTTGAGGGCCACAGTGTCTAC	CTGTCCAATCTGAACACAACTAAAAC	199	152
MID0231	2	68.0	TGTGGCTCCGCCTTCTCC	GAAAGAATGTTAAGTGTAAAGTAACTTTTAG	160	137
MID0220	2	78.8	AGGCCCACATAGAGGCCAAC	TCTGATGTCCCCTCATTCTCTG	150	155
MID0311	3	0.0	CATGACGTAGAAAAAAGTGACGTG	TTTCTGGCTTCTTATGGGATTCA	126	114
MID0321	3	11.8	TCCCTCACTGCACGGTGT	AAGAGATACTTTTGTAATTACAAATGAGG	103	89
MID0316	3	31.8	GTTTTTGGTCTTTGATTTTACATCAGT	ATCACCACAGGATGATGATGAA	103	91
MID0322	3	41.9	CATGACATAACTGACACAAACCAAAC	CCCCTTGGCTGTATTTCCTG	205	181

MID0323	3	56.6	AAACTCCTTCGTGCCGTG	TTGTGTTCCCAGCATGAAAG	104	107
MID0318	3	69.3	CAGGAGAACAAAGACCAAGATCTG	TCCTCTGAAGACCTGAGACG	184	148
MID0421	4	0.0	TCATTCTTGTTTGTAAAATGCTTG	AGCCGCTCCATACCCCCCTA	168	146
MID0412	4	12.7	CCACTGATTAAAATCACTTGTTCTGTAC	CAAAGCCATTTTTGGTATTTTTTTC	133	109
MID0414	4	23.6	TTGCTGCATAAACTACAATTTTAATC	CAAAGTGGTTTGCAGACAGACA	143	128
MID0425	4	35.8	AGCTTTTTCAAGTTTTAACCTTAAATTATT	ACAGAGAAACAAAGACCTCAAGTCTG	195	174
MID0424	4	46.2	ATATCAACTGTCATAAACATCAAAGG	GACATGTTAGTATGTTTAGAGATCACAAC	100	85
MID0428	4	53.4	AATGAAGATTCTGCCAAAAGAG	AATGAAAATATTTGTTTATATTATTCCTTG	100	88
MID0418	4	64.4	TGTTCTCAAACTGCAGCAGG	GAGGAGTCCAGGTCTTACGTCG	165	135
MID0511	5	0.0	ATAAGAGAACTGGACTGAGTGAGAGTG	ATGATGACTCAGACCCACTGAGT	97, 104	85, 92
MM03D01K	5	15.1	AACAACAGTTTCACAACTCCTG	CCCATATTCCTTCTGACTGC	198	219
MID0515	5	30.5	CTGTCTTCAAAAGTTAATAGTTTTGACAG	GTCACTAAAACAGCTGAAAATGTCTC	151	132
MID0517	5	43.0	TACACCATGAAGTGGCATCATT	AAAACTATCCATCCATTCATTTTCTTAAC	161	131
MID0518K	5	52.8	TAATCTAAAAGCACCATCTCTTCTTTTAC	ACAAACTGCTCAATGAGAAAAGTACC	203	180
MM05D05K	5	67.2	CTCCATTCAGGACCACATC	ACTGAGATGGAGGTAAAGGAAC	206	182
MID0611	6	0.0	TTTAACTCTCCCTACTTAACCGCTTA	CAGAGCCAAGTTTTGCGTG	172	149
MID0602	6	13.4	TGATGGACTGAAACTCAGATGAAG	CTGCCTAAGCTCTCATGGGG	155	142
MID0614	6	26.4	AGAACGTTCACACTGTGGCTC	GCACTTGCACTTTGGAAAGG	197	159
MID0623	6	36.2	CTGGTGCTGCTGCTTCTT	CTGCAATCCTCTGCATGTC	200	174
MID0616	6	59.4	GCTTTATACTAGTCATTACACACACCACA	CACACTTAGAAACGGCTTTAAAACA	270	258
MID0711	7	0.0	CCCCCAGATCAGCAGCAG	TACAATTATCGCCTTTGCACC	144	123

MID0722	7	14.3	TTTCTCCCTCACAACACAACC	CTTTTTGGTGTTTCTAACATATTGTTTTAG	142	127
MID0723	7	25.9	GTGTTTGATTGCTTGATTGTGT	AGTTCAAACCTTCGTAGAAAAATAAG	150	131
MID0712	7	31.9	TTCATCTTCCAACAATCATTGCT	CAGACCTTCACACTTCACAATGAC	105	94
MID0703	7	38.8	TGTAAGTGGGAAAATAATAAGCATATAGG	TTTAGAAAACACAGCTCTCTGAGAAAAG	199	179
MID0714	7	48.0	TGTATAGAAAAGAGCACAAAAGCACA	GTTGTTTGTGCCAAACATGAAG	140	122
MID0706	7	60.6	ACATTATATGGATTCCTTAATGAATGTCTT	AAACTGACCAGTAGAATACTTCAGCTTATC	152	133
MID0811	8	0.0	CCGCCTAAGTCTGGGTCC	TGCAGAGTACGATTTCAGCATATC	142	120
MID0812	8	17.2	TTATGCAAACATTTTCAGTAAATGC	GTTTCTGTGACTGCATTTCTGAAG	138	116
MID0821	8	26.0	AGGATCCTGCAAAAGTGCAA	CGTGTTTTTGAATGACTTATCACA	195	159
MID0826	8	39.6	TGTAAAAACCTAAAGTTGTTACTCAATAA	AGATTAAATCCTGCAATAGTTGGTA	139	120
MID0823	8	55.6	TAATCCGTAGATCGTCTGGTAATCTC	GCTTTGGTTCAGTTTGTTCATAAAG	142	130
MID0911	9	0.0	TCTGGACGCAGATTTTTGTCC	TTTCCTTTCACCTGTGATGACG	153	136
MID0923	9	9.1	ACACGTGAGCTAAAGCTCTGG	GACTTTGGGACTTGATTGTAACA	128	115
MID0921	9	18.8	CACGAGGCAGTCAGAGTCC	TACCAAACTGTAAAAACTGTTTCACG	105	90
MID0913	9	25.4	CCTGTGACATTTACCACATGTGTG	AAAAACATTTTCTTTAACAACTTTTTTCTC	101	85
MID0914	9	33.9	GGACACTGTGATTGGCATAGTTTTG	GTTAAATCACAAACCAAGTCCAATG	101	87
MID0916	9	46.5	AAAGTCGCAAACAAGGTTGG	AGTCAGATAAATCTGGACTTTATAACACG	100	84
MID0922	9	61.4	GTTAGGGCTCGCTGGTTTG	GGAGGCCATGGAGTGGTT	114	101
MID1011	10	0.0	CAGGCAGATGAAGACTCTGCAG	TAGGCAGTTGTTGATAAACTGGG	149	131
MID1025	10	10.5	CATAGGTGAGTATAATTTATAATATCCTGAC	TCATGAAAATGGAACGTACCA	84	93
MID1024	10	20.6	CAGCCAAAAACAGAGGAGAG	GGCACCTAATTGGACATTGT	144	136

MID1014	10	39.1	TGTCCCCAAACTGCTGAACATC	GGAATTCCTACTGAAGAATAAAATGAAAAAC	98	84
MID1028	10	48.6	TGGTGCTTGTGGAGCAAAG	CAACCGGGACAAGGAGTG	156	131
MID1015	10	64.3	CTGCTTGTCGTACAGCTGCG	GTTCCTGCAGAACCGGG	154	145
MID1121	11	0.0	GATGAAACGTCTTTGGCACTC	AGCAGAGGTGAAAGGTGGATG	123	133
MID1125	11	6.0	GGCTCCCAGGTGGCAAC	TCTGCCCATGCAGAGACG	106	97
MID1123	11	24.1	ACTTTAGCCGATCCTTCTAATAATTAC	ATCTTGTCATTTTATCTCCATAGC	101	92
MID1112	11	29.8	ACCTTCAGTTTGTACTTTTAAGCAGATTC	CTGGATTTGATCTTTTGATCTTAATTTC	149	115
MID1113	11	39.3	TGTTTTTGTTAACAAAACGTCACATT	AGCCTATGACCTTCACCCG	152	123
MID1116	11	51.6	CTCGCGTCTGAATTTTTGGG	CAGGACAAAGAAGATGTTACTGACTATG	139	123
MID1211	12	0.0	TGGAGGTAAAGAAAAACATTTCCC	GATCAGCTCATCTAAGTCATGCTAAG	150	118
MID1213	12	17.7	AGGTAAAATAAAACTAACTTCCTCTTACTATG	AGATGTCCCAGCATCCTTTG	147	125
MID1221	12	31.4	TGCTTAAAGACCCACTCCAATG	GACCCACAGCAATTTGAATG	106	96
MID1207	12	48.3	GGACACAAATCTGTTAGTTTGGG	TTTGGCTTCCAGGGAAATAGATTAG	147	133
MID1218	12	60.2	AGATTGCTTCAACAGGATTGGT	AAATAAAATCACAAACTGATGATTTATCAC	136	115
MID1311	13	0.0	TTTTTCAGAAGTTAAGAGTTTTGTTCAG	AGAACGATACGCATGAGTATCTGC	150	131
MID1324	13	10.4	CTGCATCTTTAGGGGAGCG	CCGCTTACAGATACTTCACAGTAC	110	95
MID1322	13	23.0	AACTAAATTTTTACATTCTGAACAGAAAC	TTAGAAAAATAAATTGAATTCAATCCAT	146	134
MID1314	13	33.2	TTCCTGAATTATTGATCCTGACAA	GTCAAATGGCTAAACTAGACTGTCTG	103	90
MID1306	13	44.0	GCCTTTCTTCAAATCCACTGC	GGAAAACAAAATATTAACACAGAAGCAT	161	142
MID1317	13	51.9	GCATTCACCAACAACTTGTCATG	TCTTCTCCAACTGAAGCCACTC	163	148
MID1308	13	57.6	GGCCCTTGAGACAGTAGCACTTG	AGGATGGACGCACAGAGACAGA	155	138

MID1423	14	0.0	GTTCCTACAAAGGTTGTCTTGGTG	CTTTGACTAAACTGATTAAAAGGCAG	187	170
MID1414	14	17.4	GCTGCAGATTTCCTGTTGAGAC	GGCCAAAAGTAGACTTTTATATTCTACTGG	123	110
MID1425	14	31.7	GACAGTGAGGCGTGACGA	TCCTTTAGGTTTTGGTGCCTC	107	97
MID1424	14	39.8	ACCAGAGGTTCTGTGGCAAAG	AATTTGTTTCCAAACCAACTGATG	99	85
MID1416	14	58.2	CATCTTCATGATCAATCATTTGACC	CACTGCTGGAAGTTTGGATGAAG	202	157
MID1511	15	0.0	CCTGCAAACTGTTTCACTCGC	ACGCAAAGGCTGCATCTC	112	100
MID1513	15	9.2	GCAGGATATCCTTTGAAGAGTTCTG	CTTCCTTTGGGTCCAACACC	154	138
MID1505	15	19.8	GTGTTTGTGAAACTCCTTTAAAGAGAAAAC	CCACATTAGTGGAGAACAGGATG	193	173
MID1516	15	32.8	CATCTGCACTTTATGAAACATGCT	ATGCAGGCGTCATGGTCA	149	136
MID1523	15	35.7	TGGTAAATCTGCAGAAACTTTAATATG	AGGAAGCATCAAGGAAAATCTTAG	199	169
MID1522	15	45.8	GTTCTTTCTATGTTGTTTGTGCATG	GGCCAACATTTATGCTATAAAACAAC	167	150
MID1518	15	51.5	CAGGCCTGTTCCACATGAC	GAAGATCAATCCATATTATTCACCTG	151	133
MID1611	16	0.0	CCTTTCAGCATTGAAACTTATGAAC	CGATAACGATAAATGTGCGATTTG	149	136
MID1626	16	21.5	TTTTTTTATCTTAAATGAGAAGAAAGC	CAAGGCAGCAACTGAACA	199	163
MID1603	16	33.0	AGTTTTGCTTTAGCTAAAACAAACAGG	ATGTTTTTGCTAATATTTTCTGGAGAGAG	201	186
MID1615	16	46.4	GTTTTATTTTCTGGTGTGTACCCATAG	TGGAGTTACAATAACTGGAAAAATGAC	144	123
MID1616	16	68.6	TTTCAGGTAAACCGTCCATCTG	GCCGCCTTATACTTCTTATCAAAG	189	162
MID1721	17	0.0	TGTTCTTCAAAAAACAGAAAAAGTGG	GCAAAAACACCAACTAAAAACACC	149	134
MID1715	17	13.0	TTTTTACACTAGCATGGCAGTCTC	TCACAGAGATGTGTTGGAACCA	103	91
MID1717	17	28.4	ATCCAGATTTTTGATCAACAATAGAAAC	GCCCGGCACCAACAAAGTG	103	89
MID1718	17	42.8	AGAAGTCTGCATCTCTAGATTTGACTTT	GGACGTGCCAAAAACACG	152	139

MID1725	17	51.3	AGGACTCAGGCTTTAGAAACACC	TTGATAAATGCACTACCTTTCTTTATTG	102	91
MID1719	17	62.6	TTCAGCTGAGGAGCTCCG	CTCCCTTCAACAATCTGAGAGC	203	178
MID1830	18	0.0	CAACCTGAAACACACAACAGC	ATCAGATGACAACACCTGACAGAG	150	135
MID1812	18	6.9	AAAAAAACAAATACAAATGATCTCATTG	TTAAAGTTGACCCCTAATTTAGCTTTT	139	121
MID1814	18	19.2	GTTTGTGTTTCAGTCAAAAGCATG	GGAAAAGGCCAGGGCTCT	151	134
MID1806	18	31.5	CCTAAGGGCTGACGGAGGAG	AAAAACAAGGAACTTGCCCCG	156	136
MID1807	18	39.2	CTTTATCTCTCTTCCATAAGGTAAATCAGC	AAAACCTCTAAGAGCCCGTAAATCC	204	180
MID1808	18	53.8	CTGGATTCATGAGATCATTCCGTC	TGGGACAGCAAGTCAGAGATGG	154	135
MID1921	19	0.0	TGCGGCCCACGGAGGAGG	AGCACACAGGCACACGCG	156	138
MID1924	19	11.6	CACCCGTAACTGAGCTCTCTG	GCAGGTTGACAAAGATCGTG	106	96
MID1923	19	24.3	GTTGAGCTGGCCTTATGCTC	TTAAAAAGCCTCACTTTCGAGTTC	168	146
MID1926	19	35.9	CAAATTAAATCAAGAATAAGTGGACC	CTTTCTTTTAGGCTAAACTCTCTTTG	118	106
MID1917	19	54.1	TAACTGTATCCCAACAACAGCTG	ACAAATTGAGTGCAGACTGAGTG	199	165
MID2011	20	0.0	CATGTCCCCGTAGAGCTTGT	GTGTGGCAAACATGGACCA	105	92
MID2014	20	16.5	GCTAGTGAAGCATAAACCTGGTG	GTCAAATACGTATTTACATGTAGGCCT	104	94
MID2016	20	26.3	TGGAAAATCAACTCTTTGAAAAGAAG	TTAATTTTGCTAAAGGAATGAGGTTACA	126	111
G042S318	20	37.2	CATAAACTAGACACTTGCGTTGTG	CCTTACCGATGCCTTTGTGG	168	156
MID2017	20	66.0	TGCAACATCTTTCCAACTTGG	TTTTCTAATCTGAATAACTGCAGTGG	149	135
MID2101	21	0.0	GCAGCAGTAGATCTCAGGTCCATC	TCTGCGTCCTGATTGGCTGTAG	156	141
MID2122	21	4.0	TTCACTGTCTAGTGGGTCTAGATG	TTTTTTCTATAACCATGGCATATG	105	96
MID2112	21	18.7	CTGCAGAGACTCTAGAACCTCCA	TCTCTGCAGGCCTTGAACTT	155	125

MID2113	21	36.5	TTTTACCTAAATTTAGGGTTTTCTTTCC	CATTTACAACAAAACAGATGGAGC	152	132
MID2126	21	49.4	GAATGAAAAGGGATTTTACGTTC	TTCTTCTGGAAACAGTGCCA	156	135
MID2124	21	59.6	GATGTCGTCCACTTGCTTG	TCTCCCAGGTTATGTAATAACCA	106	93
MID2116	21	65.9	GTGTTAAATGAAACCTTGTGAAAAGTT	TTTGCATCCGTACAATATGTGC	152	129
G021S407	21	70.7	AGTACCTGATCCACACAAGCAG	GGATGTGGAGTTGGATCAGCA	178	190
MID2221	22	0.0	ACTGCCCAATCGGGATG	AGGGAGAACAGATTTCATTCATG	148	134
MID2211	22	16.6	TCATAAATACATTTCTGGATCAGGC	AGTAAAAAGGTCTAGCTGCTGTTACCT	196	152
MID2222	22	31.7	GATTAACTTAACTACCATTTTATTTACCG	ATGACAAAAACTTGTCTTTTGCTAAG	128	116
MID2212	22	36.5	GGCTATGTGACTGGAACTTCATTC	TATTGTGGAGCTTTTAATTATTACAACGTC	194	148
MID2213	22	54.6	TTTTTATGTTTTGTCCTGTTTAAATTCAG	TGTGAAGTGGATTTTTTGGAGAAC	150	135
MID2216	22	61.5	AGCTTTATGAAATTGGACTTGAAATG	TTCACAAACTAGCATGCTTTCAAT	150	133
MID2311	23	0.0	GGTTCACTTTAAGCACCAAATGTG	TTTGCTGCATGGCTCATCT	206	170
MID2322	23	16.5	TGTCCTTGATGTTGGAAAACAT	TCCTCTGAAGCATATCTACTTCAATC	143	133
MID2312	23	23.7	CTTTTTCTCTCACGTCTTTACAATGC	CATTTACTTCTATTAACGTTTTCCTTTTTA	113	100
MID2313	23	34.2	GGCTCCTCTAATAGATGCTGAACC	ATATAGGGTAGTTGTATCGGCCATG	105	92
MM05G07K	23	50.6	ACATCACAAAGTGAGAACAGC	CATCAGCACTTGAAAGCG	148	140
MID2321	23	56.3	AAACGGCAGCACAAATGTC	AATTGTTGTGGTTTTGGACTTTC	102	94
MID2305	23	64.1	CATCGAGAAATCCTCGGTAACA	TGGCCAGTGTACTAATTGTTTGC	203	239
MID2411	24	0.0	GTTCGTCATCACCTATCAAAGGAG	AATGACATGGCAAGCAACCA	198	162
MID2428	24	10.8	TATTTTATTAACACATTTGATTTGGC	AGGGAGGTGGAGGAGGTG	141	122
MID2412	24	19.0	CAGTTCTTATTTCATTGCAGTCAAAG	AGAATTCCAGACATCTAAGTAAATTTGTG	150	120

MID2414	24	30.9	TAAGGGCACCATACGACAGC	CCAAAATGTTGCTGTTTTTAGCA	108	96
MID2421	24	38.8	AAAAATGTTTATTTGCATGTTGATTG	AGCTGTTGTTCATATTAAAACTCAAAGAC	154	136
MID2425	24	54.7	GTGGGAGCAAAGACTCATGG	AGGTGAGAGCAGTCCAGCC	106	95

Position indicates a map distance. The map function is Haldane. All amplicon size were decided by MultiNA.
