# Supplementary material for: Genetic Analysis of Vertebral Regionalization and Number in Medaka (Oryzias latipes) Inbred Lines
Source: G3 (Bethesda). 2012 Nov 1;2(11):1317–23. doi: 10.1534/g3.112.003236 (PMC3484662; doi:10.1534/g3.112.003236)
Supplement: Supporting Information [file supp_2.11.1317_FigureS1.pdf]

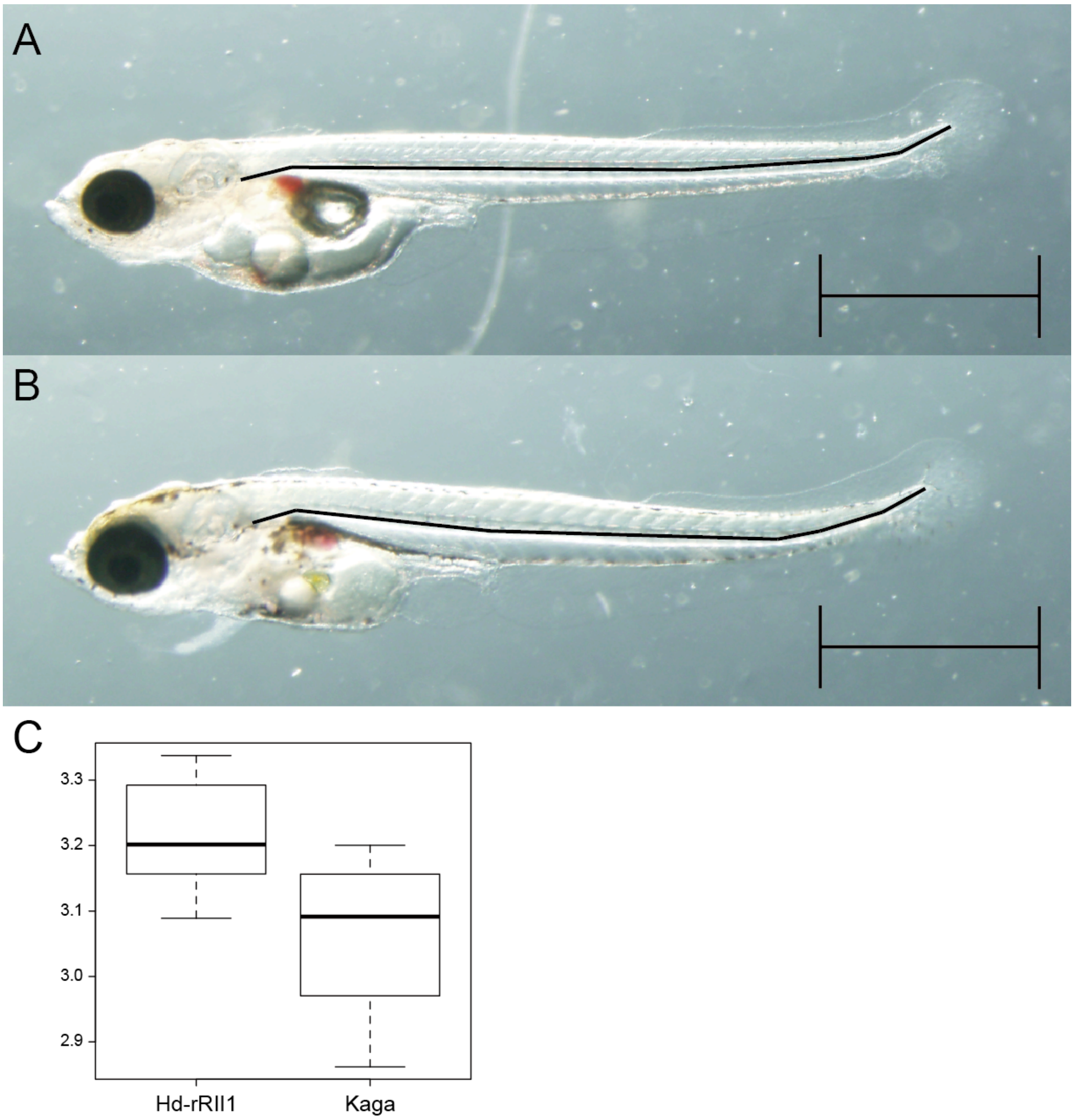

Figure S1 Measure of fry of Hd-rRII1 and Kaga. Photo is seven days fry of Hd-rRII1 (A) and Kaga (B). The scale bars represent 1 mm. Straight line was drawn along with notochord and it was considered as trunk length. The length was measured in imageJ software. (C) Boxplot of the trunk length. The mean ± S.E.M. of the trunk length of are 3.22 ± 0.02 mm in Hd-rRII1 (n = 16), and 3.06 ± 0.04 mm in Kaga (n = 11), respectively. Hd-rRII1 is longer than Kaga in this stage. The trunk length showed significant difference as judged by Welch's *t* test ($p = 0.002$).
